# Type-specific persistence, clearance and incidence of high-risk HPV among screen-positive Rwandan women living with HIV

**DOI:** 10.1186/s13027-021-00355-6

**Published:** 2021-02-19

**Authors:** Gad Murenzi, Patrick Tuyisenge, Faustin Kanyabwisha, Athanase Munyaneza, Benjamin Muhoza, Gallican Kubwimana, Anthere Murangwa, Leon Mutesa, Kathryn Anastos, Philip E. Castle

**Affiliations:** 1 Rwanda Military Hospital, Kigali, Rwanda; 2 Division of Cancer Prevention, National Cancer Institute, Rockville, MD USA; 3 Research for Development (RD Rwanda), Kigali, Rwanda; 4 Center for Human Genetics, College of Medicine and Health Sciences, University of Rwanda, Kigali, Rwanda; 5 Albert Einstein College of Medicine, Bronx, NY USA; 6 Division of Cancer Epidemiology and Genetics, National Cancer Institute, Rockville, MD USA

**Keywords:** Hr-HPV persistence, Incidence, HIV, HPV vaccine

## Abstract

**Background:**

Persistent infection with high-risk human papillomavirus (hrHPV) is a critical step in cervical carcinogenesis. We report on type-specific hrHPV persistence, clearance and incidence among screen-positive Rwandan women living with HIV (WLWH).

**Methods:**

This was a nested analysis from a large cervical cancer screening study of ~ 5000 Rwandan WLWH. Women who tested positive for hrHPV and/or visual inspection with acetic acid were referred to colposcopy. For a subset of women (*n* = 298) who were ≥ 6 months delayed in receiving colposcopy, we tested their screening and colposcopy visit specimens using the AmpFire HPV genotyping assay that tests 14 hrHPV types (16, 18, 31, 33, 35, 39, 45, 51, 52, 56, 58, 59, 66, and 68) individually.

**Results:**

The mean, median (interquartile range [IQR]) and range of time between the screening and colposcopy visits were 644, 650 (490–820.5) and 197–1161 days, respectively. Mean, median (IQR) and range of age at the screening visit were 38, 37 (34–43) and 30–54 years, respectively. Two-hundred eighty-three (95.0%) had CD4 count (cells per mm^3^) data available at baseline with mean, median (IQR) and range of 592, 513 (346–717) and 0–7290, respectively. Two-hundred thirty-five WLWH were positive for at least one hrHPV type at the screening visit, of whom 50.2% had at least one HPV type-specific infection persist; 37.2% of all HPV infections detected at the screening visit persisted. Compared to all other HPV types in aggregate, HPV16 (vs. non-HPV16 types) (47.7%, *p* = 0.03) and HPV33 (vs. non-HPV33 types) (56.7%, p = 0.03) were significantly more likely, and HPV39 (vs. non-HPV39 types) (6.7%, *p* = 0.01), HPV51 (vs. non-HPV51 types) (15.6%, *p* < 0.01), and HPV66 (vs. non-HPV66 types (17.9%, *p* = 0.04) were significantly less likely, to persist. Lower CD4 counts were associated with having any persistent hrHPV infection (p_trend_ = 0.04) and multiple persistent hrHPV infections (p_trend_ = 0.04).

**Conclusion:**

There is a significant proportion of WLWH with persistent hrHPV infection, emphasizing the need to vaccinate them against HPV prior to becoming sexually active.

**Supplementary Information:**

The online version contains supplementary material available at 10.1186/s13027-021-00355-6.

## Introduction

Cervical cancer is the fourth most common cancer among women and the fourth leading cause of female cancer deaths worldwide with 570,000 new cases and 311,000 deaths occurring in 2018 [1]. Africa, especially Sub-Saharan Africa (SSA), bears the greatest burden of cervical cancer with incidence and mortality rates in Southern, Eastern and Western Africa 7–10 times those in North America, Australia/New Zealand and Western Asia [1]. This high burden of cervical cancer is in part due to the high prevalence of human immunodeficiency virus (HIV) infection and acquired immunodeficiency syndrome (AIDS). SSA is home to almost 70% of the nearly 38 million people living with HIV (PLWH) globally [2]. Women living with HIV (WLWH) bear an even greater burden of cervical cancer compared to their HIV-negative counterparts [3–5] and cervical cancer was included as an AIDS defining illness in 1993 [6].

Infection with high-risk human papillomavirus (hrHPV), the necessary cause of virtually all cervical cancer, is a very common sexually transmitted infection [7]. Most sexually active individuals acquire it at some point during their lifetime [7]. Vaccination against HPV infection is the most important primary prevention strategy for the prevention of cervical cancer. A recent study done in Sweden provided the first long awaited evidence that HPV vaccination reduces the incidence of invasive cervical cancer among females, with the greatest reductions observed in those who had been vaccinated when under the age of 17 years [8].

Recent reports indicate that 127 of 225 countries and territories globally have national HPV vaccination programs and that 41 additional countries and territories are set to introduce the vaccine by the end of 2023 [9]. The World Health Organization (WHO) recommends HPV vaccination for all women aged 9–14 years with 2 doses of either the Cervarix (2vHPV) with protection against HPV 16 and 18 or Gardasil (4vHPV) with protection against HPV 6, 11, 16 and 18 and with 3 doses for women beginning the series at 15 years and older and for those known to be immunocompromised and/or living with HIV [10]. As of late 2016, only Gardasil-9 (9vHPV) with protection against the same types as 4vHPV but with five additional high-risk types (HPV 31, 33, 45, 52 and 58) is being distributed in the United States (US) and is recommended as 2 doses for girls 11–12 years with catch up until 26 years. The recommendations were recently updated to include HPV vaccination to up to 45 years with shared clinical decision-making after risk assessment for both women and men [11].

Yet, many women have already been exposed to HPV, and HPV vaccination does not treat pre-existing HPV infections or related abnormalities. Most hrHPV infections clear/become undetectable within a year or two [12–15]. Persistent hrHPV infection is a critical step in cervical carcinogenesis [16]. hrHPV persistence for a year [17] or two [18] strongly predicts having or developing cervical precancer and cancer. Thus, factors that increase the likelihood of HPV persistence increase the risk of cervical precancer and cancer. WLWH, because of an impaired immune response, are more likely than HIV-negative women to have a persisting HPV infection [19–28]. Among WLWH, higher CD4 count [19, 27, 29–32], lower HIV viral load [30, 31, 33] and/or using antiretroviral therapy (ART) [21, 33, 34] are associated with an increased likelihood of HPV clearance.

There are few data on the natural history of type-specific hrHPV infection in SSA, especially among WLWH. Adler et al. found that 12-month HPV genotype-specific persistence was almost 8-fold more likely in young (aged 17–21 years) WLWH (31%) compared to young HIV-negative women (4%) [28]. A study of Nigerian women found WLWH to have higher prevalence, persistence, and presence of multiple HPV infections compared to HIV-negative women [35]. Another study found 18-month hrHPV persistence of 51.5% in WLWH from Burkina Faso (median age = 36 years) and 44.7% in WLWH from South Africa (median age = 34 years) [33]. One study conducted in Rwanda reported a 24-month hrHPV persistence of 56% in WLWH compared to 0% in HIV-negative women [22].

We recently reported that the prevalence of hrHPV prevalence among Rwandan WLWH has been decreasing over the past 12 years most likely due to improving HIV care and treatment over time with most women (over 95%) taking antiretroviral therapy (ART) [36]. That study also showed that the trend of hrHPV prevalence increases with decreasing CD4 cell count indicating the role of immunity in HPV prevalence and perhaps persistence and progression to precancer and cancer. Given the relationship of prevalence with persistence (Prevalence = Incidence x Duration [Persistence]), we investigated the short-term, type-specific hrHPV persistence among screen-positive Rwandan WLWH.

## Methods

### Study design, population and setting

We conducted a nested analysis from a large cervical cancer screening study of ~ 5000 Rwandan WLWH. The protocol of the parent study has been previously described in detail [37]. In brief, WLWH were screened for cervical cancer using different screening methods. Following a positive screening test (positive for hrHPV by the Xpert HPV test (Cepheid, Sunnyvale, CA, USA) and/or by visual inspection with acetic acid-VIA), women were referred for rigorous colposcopic evaluation, including a 4-quadrant microbiopsy/biopsy protocol and specimen collection for biomarker testing.

The study was approved by relevant ethics committees and all participants provided written informed consent.

### Laboratory testing

A subset of women (*n* = 298) who were 6 to 38 months delayed in receiving colposcopy (77.0% hrHPV positive only, 15.9% VIA positive only, and 7.1% hrHPV and VIA positive). Their screening- and colposcopy-visit specimens were tested for hrHPV using both the Xpert HPV test and the AmpFire HPV genotyping assay, which tests for 15 HPV types, including 14 hrHPV types (16, 18, 31, 33, 35, 39, 45, 51, 52, 56, 58, 59, 66, and 68) that are also detected by the Xpert assay.

The AmpFire HPV genotyping assay (Atila Biosystems Inc., Mountain View, CA, USA) is an isothermal nucleic acid amplification-based, real-time fluorescence detection of 15 HPV genotypes (16, 18, 31, 33, 35, 39, 45, 51, 52, 53, 56, 58, 59, 66, and 68) individually in 4 reaction tubes. Testing was done according to the manufacturer’s protocol. Briefly, an aliquot of the stored specimen in PreservCyt solution was pelleted by centrifugation, the supernatant decanted and pelleted cells suspended in lysis buffer. The cell suspension incubated for 10 min at 95 °C to lyse the cells. For each reaction, 2 μL of lysate was mixed with 12 μL of Reaction Mix and 11 μL of one of the four Reaction Mixes. The resulting four reaction tubes for every sample were incubated in Powergene 9600 fluorescence real-time polymerase chain reaction (PCR) system at 60^o^ C with fluorescence from FAM/HEX/ROX/CY5 channels measured every minute.

After running for approximately one (1) hour, the amplification results were interpreted according to exponential curves developed during the process. If negative control showed no exponential curves and positive control showed exponential curves, this experiment run was valid. The next step was to examine the set of four tubes corresponding to a specimen. Multiplex HPV infections could result in multiple exponential curves for a specimen. If no exponential curve other than internal control (Hex channel in PM-3 tube) was present for a sample, this sample was negative. If there was no exponential amplification curve in any of four tubes or any fluorescence channels, the sample failed the test. A failed sample usually indicated not enough DNA in the sample and it was reprocessed [38].

The Xpert HPV assay is a qualitative, real-time PCR assay for the detection of hrHPV DNA. The Xpert HPV assay includes simultaneous detection of 14 hrHPV types, hydroxymethylbilane synthase (HMBS) and an internal Probe Check Control. The 14 targeted hrHPV types are detected in five fluorescent channels: (1) HPV16, (2) HPV18 and HPV45, (3) HPV31, 33, 35, 52 and 58, (4) HPV51 and HPV59 and (5) HPV39, 56, 66 and 68. HMBS (fluorescent channel 6) verifies specimen adequacy. Specimens were mixed and a 1 mL pre-aliquot was removed using a disposable pipette and placed in the testing cartridge per the manufacturer’s instructions. Unsatisfactory results due to insufficient cellular content were retested. If the second test was also unsatisfactory, the final result was being recorded as unsatisfactory [37].

HIV data including CD4 cell count, viral load results and ART data were extracted from the patient electronic medical records (OpenMRS) as indicated in the study protocol.

### Statistical analysis

We compared baseline characteristics for women included in this analysis to those not included. Age and time between visits were categorized as quartiles, CD4 cell count was stratified by standard clinical categories and viral load was categorized as detectable vs. undetectable with < 200 copies/mL being undetectable according to Rwandan guidelines. We examined the outcomes (e.g., persistence, clearance, and incidence) of individual hrHPV infections as detected by AmpFire. Each woman contributed to multiple outcomes given that each woman could be infected with multiple hrHPV types. We categorized the women according to the following hierarchy to account for multiple HPV infections: persistence>clearance>incidence>negative. The relationship of various independent variables with persistence and clearance of hrHPV infection (the outcome variable) was tested using the Chi square and Fisher’s exact tests accordingly. The exact version of the McNemar’s Chi square test was used to compare persistence, incidence and clearance as well as those who remained negative for each of the 14 hrHPV types individually by the AmpFire assay. Finally, a logistic regression model was put together to test the association between the independent variables of known association and the outcome variable. Unadjusted or crude odds ratios (OR) and adjusted odds ratios (aOR) with their 95% confidence intervals (95% CI) were used as measures of association. *P* values < 0.05 were considered statistically significant.

## Results

Descriptive statistics of the screen-positive women included in this analysis because of late colposcopy (*n* = 298) and those excluded from this analysis because they underwent colposcopy in < 6 months (*n* = 1116) are shown in Supplemental Table 1. Women included in this analysis were younger (*p* < 0.01), started having sex at an earlier age (*p* < 0.01), were more likely screen HPV positive (*p* = 0.01) but less likely to screen VIA positive (*p* < 0.01), and more likely to have a high-grade colposcopic impression (*p* < 0.01) compared to women excluded from this analysis.

Among the screen-positive women who did not undergo colposcopy for 6 months or more and included in this analysis, the mean, median (interquartile range [IQR]) and range of time between the screening and colposcopy visits were 644, 650 (490–820.5) and 197–1161 days, respectively. Mean, median (IQR) and range of age at the screening visit were 38, 37 (34–43), and 30–54 years, respectively. Two-hundred eighty-three (95.0%) had CD4 count data available at baseline, with mean, median (IQR) and range of 592, 513 (346–717) and 0–7290 per mm^3^, respectively. Table 1 shows additional participant characteristics for all 298 participants as well as a comparison of the 235 women who tested positive for one or more hrHPV type who cleared any hrHPV infection versus those who had at least one persistent hrHPV infection.

**Table 1:** General participant characteristics and results by persistence vs. clearance of hrHPV by AmpFire

Characteristic	All Women	Clearance	Persistence	p*
	***n*** = 298	***n*** = 117	***n*** = 118	
	n (%col)	n (%col)	n (%col)	
**Age Group (Years)**				
30–33	70 (23.5)	31 (26.5)	23 (19.5)	0.19
34–36	60 (20.1)	25 (21.4)	28 (23.7)	
37–42	82 (27.5)	35 (29.9)	28 (23.7)	
43–54	85 (28.5)	26 (22.2)	39 (33.0)	
Missing	1 (0.3)	0 (0.0)	0 (0.0)	
**Age at Sexual Initiation (Years)**				
< 18	180 (60.4)	51 (43.6)	42 (35.6)	0.21
≥ 18	118 (39.6)	66 (56.4)	76 (64.4)	
**Number of Sexual Partners (Lifetime)**				
≤ 5	245 (82.2)	94 (80.3)	98 (83.1)	0.59
≥ 6	53 (17.8)	23 (19.7)	20 (16.9)	
**Number of Sexual Partners (Last 6 Months)**				
≤ 5	285 (95.6)	111 (94.8)	113 (95.8)	0.90
≥ 6	8 (2.7)	3 (2.6)	3 (2.5)	
Missing	5 (1.7)	3 (2.6)	2 (1.7)	
**Number of Children**				
< 5	230 (77.2)	90 (76.9)	92 (78.0)	0.85
≥ 5	68 (22.8)	27 (23.1)	26 (22.0)	
**CD4 (per mm**^**3**^**) (Screening Visit)**				
≥ 500 (ref)	146 (49.0)	62 (53.0)	49 (41.5)	0.34
350–499	64 (21.5)	23 (19.7	26 (22.0)	
200–349	48 (16.1)	17 (14.5)	22 (18.6)	
< 200	25 (8.4)	8 (6.8)	15 (12.7)	
Missing	15 (5.0)	7 (6.0)	6 (5.1)	
**On Anti-Retrovirals**				
No	3 (1.0)	1 (0.9)	2 (1.7)	1.00
Yes	293 (98.3)	115 (99.3)	115 (97.5)	
Missing	2 (0.7)	1 (0.9)	1 (0.9)	
**HIV Viral Load**^**a**^ **(Screening Visit)**				
Undetectable	236 (79.2)	91 (77.8)	92 (80.0)	**0.04**
Detectable	28 (9.4)	9 (7.7)	18 (15.3)	
Missing	34 (11.4)	17 (14.5)	8 (6.8)	
**Time between Screening and Colposcopy Visits (Days)**				
197–489	72 (24.2)	27 (23.1)	40 (33.9)	0.06
490–649	73 (24.5)	39 (33.3)	22 (18.6)	
650–820	74 (24.8)	22 (18.8)	29 (24.6)	
821–1161	73 (24.5)	25 (21.4)	25 (21.9)	
Missing	6 (2.0)	4 (3.4)	2 (1.7)	
**VIA Result (Screening Visit)**				
Negative	228 (76.5)	100 (85.5)	104 (88.1)	0.50
Positive	68 (22.8)	15 (12.8)	14 (11.9)	
Missing	2 (0.7)	2 (1.7)	0 (0.0)	
**Number of hrHPV Types (Screening Visit)**				
0	63 (21.1)	–	–	1.00
1	133 (44.6)	66 (56.4)	67 (56.8)	
≥ 2	102 (34.2)	51 (43.6)	51 (43.2)	
**Colposcopic Impression**				
Low-Grade	266 (89.3)	111 (94.9)	96 (81.4)	**< 0.01**
High-Grade	25 (8.4)	1 (0.9)	20 (17.0)	
Missing	7 (2.4)	5 (4.3)	2 (1.7)	

*Fisher’s Exact to compare persistence vs. clearance

^a^Viral load was categorized as detectable and undetectable according to the Rwanda guidelines where all viral load results of less than 200 viral copies/mL of blood

Table 2 shows the hrHPV genotyping results for the screening and colposcopy visits. Clearance of hrHPV types present at baseline was more common than incidence of new hrHPV types at follow-up for all hrHPV types although not statistically significant for the rarer hrHPV types. Of the 235 women who had a hrHPV type detected at baseline, 118 (50.2%) had at least one hrHPV persistent infection.

**Table 2:** Overall and type-specific persistence, incidence and clearance hrHPV infections in 298 screen-positive WLWH

hrHPV type	Negative N (%row)	Incidence N (%row)	Clearance N (%row)	Persistence N (%row)	p*	% Persistence^**a**^	***p***^**‡**^
**Any**	43 (14.4)	20 (6.7)	117 (39.3)	118 (39.6)	< 0.01	50.2	n/a
**HPV16**	198 (66.4)	14 (4.7)	45 (15.1)	41 (13.8)	< 0.01	47.7	**0.03**
**HPV18**	262 (87.9)	5 (1.7)	16 (5.4)	15 (5.0)	0.03	48.4	0.18
**HPV31**	265 (89.0)	5 (1.7)	19 (6.4)	9 (3.0)	< 0.01	32.1	0.69
**HPV33**	263 (88.3)	5 (1.7)	13 (4.4)	17 (5.7)	0.09	56.7	**0.03**
**HPV35**	262 (87.9)	6 (2.0)	19 (6.4)	11 (3.7)	0.01	36.7	1.00
**HPV39**	277 (93.0)	6 (2.0)	14 (4.7)	1 (0.3)	0.12	6.7	**0.01**
**HPV45**	268 (89.9)	5 (1.7)	14 (4.7)	11 (3.7)	0.06	44.0	0.52
**HPV51**	261 (87.6)	6 (2.0)	27 (8.7)	5 (1.7)	< 0.01	15.6	**< 0.01**
**HPV52**	256 (85.9)	12 (4.0)	17 (5.7)	13 (4.4)	0.46	43.3	0.56
**HPV56**	258 (86.6)	7 (2.4)	25 (8.4)	8 (2.7)	< 0.01	24.2	0.13
**HPV58**	263 (88.3)	6 (2.0)	15 (5.0)	14 (4.7)	0.08	48.3	0.23
**HPV59**	282 (94.6)	5 (1.7)	8 (2.7)	3 (1.0)	0.58	27.3	0.75
**HPV66**	266 (89.3)	4 (1.3)	23 (7.7)	5 (1.7)	< 0.01	17.9	**0.04**
**HPV68**	283 (95.0)	6 (2.0)	7 (2.4)	2 (0.7)	1.00	22.2	0.49
**All HPV Infections**^**b**^	3664 (87.8)	92 (2.3)	262 (6.3)	155 (3.6)	< 0.01	37.2	n/a

^a^Summation of infection-level outcomes of all HPV types i.e., each woman can contribute 14 results for each individual high-risk HPV genotype

^b^Among women who were hrHPV positive by AmpFire at the screening visit

*Exact version of the McNemar’s chi-square test; All persistence is type-specific and women can contribute to multiple outcomes since they may have multiple infections

^**‡**^vs. persistence for all other types except that type e.g., HPV16 vs. all other (non-HPV16) hrHPV types (exact version of a Pearson chi-square test))

The 235 WLWH had 417 hrHPV types detected at the screening visit (1.77 types per woman; 133 with one hrHPV type and 102 with two or more hrHPV types), of which 155 (37.2%) HPV types persisted to the colposcopy visit. Compared to all other HPV types in aggregate, HPV16 (vs. non-HPV16 types) (47.7%, *p* = 0.03) and HPV33 (vs. non-HPV33 types) (56.7%, p = 0.03) were significantly more likely to persist. Conversely, HPV39 (vs. non-HPV39 types) (6.7%, *p* = 0.01), HPV51 (vs. non-HPV51 types) (15.6%, *p* < 0.01), and HPV66 (vs. non-HPV66 types (17.9%, *p* = 0.04) were significantly less likely to persist.

**Fig. 1:**
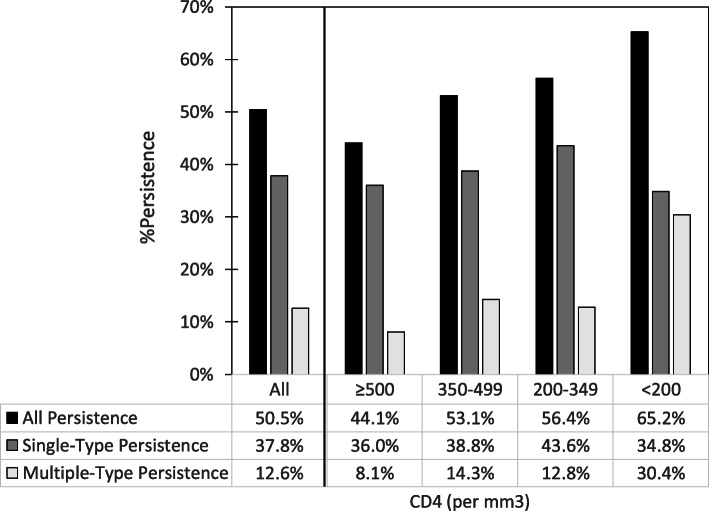
hrHPV type-specific persistence and number of hrHPV-persistent infections with baseline CD4 counts among baseline hrHPV-positive

Figure 1 shows the crude relationship of CD4 count with the likelihood of type-specific hrHPV persistence for the 222 WLWH for whom we had CD4 count data at the screening visit. Women in lower CD4 count categories had higher likelihood of any type-specific hrHPV persistence: 44.1% for those with CD4 ≥ 500/mm^3^ vs. 65.2% for those with CD4 < 200/mm^3^ (p_trend_ = 0.04). WLWH with lower CD4 counts were more likely to have multiple persistent hrHPV infections (p_trend_ = 0.01); among those who had any persistent hrHPV infection, WLWH with lower CD4 counts were marginally more likely to have multiple persistent hrHPV types (18.4% for WLWH with CD4 ≥ 500/mm^3^ vs. 46.7% for WLWH with CD4 < 200/mm^3^ (p_trend_ = 0.06). WLWH with lower CD4 counts were non-significantly more likely to have multiple hrHPV infections (p_trend_ = 0.13) and given multiple hrHPV infections, more likely to have multiple persistent hrHPV infection compared to women with higher CD4 counts.

Factors associated with hrHPV persistence are shown in Table 3. High-grade colposcopic impression (versus low-grade) at the follow-up (colposcopy) visit was most strongly associated with type-specific hrHPV persistence (aOR = 24.90, 95%CI = 3.08–201.22). Compared to WLWH aged 30–33 years at the screening visit, those aged 43–56 years were more likely to have type-specific hrHPV persistence (aOR = 2.26, 95%CI = 1.01–5.05). WLWH who came back for colposcopy in 490–649 days were less likely to have type-specific hrHPV persistence than WLWH who came back in 197–489 days (aOR = 0.35, 95%CI = 0.16–0.77) but longer interval times were not associated with the likelihood of type-specific hrHPV persistence. Finally, although there was no category of CD4 count that was associated with having a persistent type-specific hrHPV infection, there was a marginally significant trend of WLWH in lower CD4 count categories being increasingly likely to have type-specific hrHPV persistence (p_trend_ = 0.06).

**Table 3:** Factors associated with persistence (vs. clearance) of any hrHPV (*n* = 235 hrHPV+ at the screening visit)

	N (%)	OR	95%CI	aOR	95%CI
**CD4 (per mm**^**3**^**) (Screening Visit)**					
≥ 500 (ref)	111 (47.2)	1	–	1	–
350–499	49 (20.9	1.43	0.73–2.81	1.46	0.67–3.15
200–349	39 (16.6)	1.64	0.78–3.42	1.56	0.69–3.51
< 200	23 (9.8)	2.37	0.93–6.05	2.48	0.84–7.35
Missing	13 (5.5)	1.08	0.34–3.44	2.19	0.57–8.41
p_trend_^a^=		**0.04**		**0.06**	
**Detectable HIV Viral Load (Screening Visit)**					
No (ref)	183 (77.9)	1	–	1	–
Yes	27 (11.5)	1.99	0.84–4.63	1.75	0.67–4.57
Missing	25 (10.6)	0.47	0.19–1.13	**0.34**	**0.12–0.98**
**Age Group (years) (Screening Visit)**					
30–33 (ref)	54 (23.0)	1	–	1	–
34–36	53 (22.6)	1.5	0.70–3.23	1.74	0.74–4.08
37–42	63 (26.8)	1.1	0.52–2.25	0.80	0.34–1.83
43–54	65 (27.7)	2.0	0.97–4.21	**2.26**	**1.01–5.05**
p_trend_=		0.13		0.17	
**Follow-Up Time (Days)**					
197–490 (ref)	66 (28.1)	1	–	1	–
490–649	62 (26.4)	0.38	**0.29–0.79**	**0.35**	**0.16–0.77**
650–820	49 (20.9)	0.89	0.43–1.86	0.94	0.41–2.14
821–1161	52 (22.1)	0.68	0.32–1.41	0.74	0.33–1.67
Missing	6 (2.6)	0.34	0.06–1.97	0.61	0.08–4.59
p_trend_^a^=		0.68		0.93	
**Colposcopic Impression**					
low-grade (ref)	207 (88.1)	1	–	1	–
high-grade	21 (8.9)	**23.1**	**3.0–175.52**	**24.90**	**3.08–201.22**
Missing	7 (3.0)	0.86	0.66–1.14	0.33	0.05–2.11
**Number of hrHPV Types Detected at the Screening Visit**					
1 (ref)	133 (56.6)	1	–		
≥ 2	102 (43.4)	0.99	0.59–1.66		

^a^missing values excluded from tests of trend, *OR* unadjusted odds ratio, *aOR* adjusted odds ratio and *95%CI* 95% confidence interval

## Discussion

The prevalence of hrHPV infection among Rwandan WLWH has been decreasing over the past decade or so [36]. This phenomenon is most likely due to improving HIV care, treatment and management over time, including early initiation and extensive coverage (95%) of ART, which has led to improved health and immune status as reflected by higher CD4 counts [36].

However, little is known about the natural history of hrHPV infection in Rwandan WLWH, who are at increased risk of both hrHPV persistence and progression to precancer and cancer. The differences we found in the comparison of the group of screen-positive women included in this analysis with those who were not included indicate that the group included in this analysis is perhaps at increased risk of hrHPV persistence given that most of them had their first sexual intercourse at a younger age, most were hrHPV positive at baseline and most of them had high-grade lesions on colposcopy compared to the group that was not included in this analysis.

We found considerable hrHPV persistence (39.6%) and incidence (6.7%) among the 298 screen-positive Rwandan WLWH we retested for HPV after a median of 22 months of follow-up. These data provide additional evidence that WLWH are at high risk of hrHPV infection, persistence, and perhaps re-infection or reactivation. To our knowledge, this is the first study to report type-specific hrHPV persistence, incidence, and clearance among Rwandan WLWH. HPV persistence was highest for HPV16 (13.8%) followed by HPV33 (5.7%) and HPV18 (5.0%) and incidence was highest for HPV16 (4.7%) followed by HPV52 (4.0%). The second highest persistent and incident hrHPV types are both not included in the 4vHPV vaccine but rather in the 9vHPV vaccine hence the public health importance of introducing the 9vHPV vaccine in SSA in general and in Rwanda in particular. Women with higher CD4 cell counts were less likely to have a persistent hrHPV infection as well as multiple hrHPV infections most likely due to better immune responses resulting from improving HIV care and management as described above.

hrHPV persistence observed in this group of Rwandan WLWH was generally lower than among other study populations of mid-adult WLWH living in SSA: others have reported 24-month hrHPV persistence of 56% in Rwanda [22] and 18-month hrHPV persistence of 51.5% in Burkina Faso and 44.7% in South Africa [33]. A study in Nigeria reported a HPV clearance rate of 1.6% per month in WLWH or a 12-, 18-, 22-, and 24-month persistence of 81, 71, 65, and 62%, respectively [35].

Differences in HIV disease status (e.g., CD4 and HIV viral load) and care (ART use) may explain differences in the measurable HPV persistence among studies. A study done among WLWH of SSA origin found that sustained viral suppression and higher CD4 cell counts were associated with a significantly decreased persistence of hrHPV infection [30]. Likewise, the HPV genotyping tests used differ between studies and differences in testing analytic sensitivity might influence HPV persistence if one test is picking up lower HPV viral load infections that are more likely to clear than persist.

Differences in analytic sensitivity for individual HPV types may influence which types were found to be more persistent. For example, we found HPV16, 33, 18, 58 and 52 to be the most persistent (in order of highest proportion) but the study among Nigerian WLWH found HPV52, 35, 31, 51 and 16/18 to be the most persistent [35]. Finally, differences in age, as a proxy for how long the prevalent HPV infection was already present, might also introduce differences in the likelihood of persistence. For example, the study by Alder et al. found that in young adult women only 31% of HPV infections persisted for 12 months [28].

In our analysis, hrHPV persistence was strongly associated with high-grade colposcopic impression as an insensitive but specific proxy for high-grade cervical abnormalities. These data are consistent with short-term hrHPV persistence being a risk factor for cervical cancer [17, 18].

Our study has some limitations. We recruited WLWH only from the capital city of Rwanda, Kigali, who may not be representative of the entire country. Moreover, those who were included were higher risk than those excluded from the analysis. All participants included in our analysis were screen-positive (all positive for either hrHPV and/or VIA). WLWH who are at very high risk of hrHPV acquisition, persistence and incidence partly explaining the high persistence rates, although these are not very different from findings from other studies.

We were not able to have women come in for several visits in order to look at the differences in persistence, incidence and clearance over time. In addition, the definition of persistence proposed by Munoz et al. where persistence is to be based on incident infection with persistent infections being those lasting more than the median duration was not taken into account in our study [39]. Finally, we did not have histologic endpoints at the time of the study, which would provide confirmation of the cervical cancer risk associated with short-term hrHPV persistence.

## Conclusions

In conclusion, we found a considerable proportion of Rwandan WLWH with persistent hrHPV infection. Women with lower CD4 cell counts were more likely to have persistent hrHPV infection, confirming the effect of immune status on HPV persistence. All seven hrHPV types in the prophylactic 9vHPV vaccine [40] were found to have considerable viral persistence, reinforcing the need to introduce this HPV vaccine for all women, especially for high-risk populations, mainly WLWH, prior to their becoming sexually active for the prevention of cervical and other HPV-related cancers.

## Supplementary Information

**Additional file 1: Supplemental Table 1.** Comparison of screen-positive women included in this analysis and those not included.

## Data Availability

The datasets used and/or analyzed during the current study are available from the corresponding author on reasonable request.

## Abbreviations

AIDS: Acquired Immunodeficiency Syndrome
aOR: Adjusted Odds Ratio
ART: Anti-retroviral Therapy
CD4: Cluster of Differentiation 4
CI: Confidence Interval
DNA: Deoxyribonucleic Acid
HIV: Human Immunodeficiency Virus
HMBS: Hydroxymethylbilane Synthase
HPV: Human Papillomavirus
hrHPV: High-risk Human Papillomavirus
IQR: Inter-quartile Range
OR: Odds Ratio
PCR: Polymerase Chain Reaction
PLWH: People Living With HIV
SSA: Sub-Saharan Africa
USA: United States of America
VIA: Visual Inspection with Acetic acid
WLWH: Women Living With HIV

## References

[1] Bray F, Ferlay J, Soerjomataram I, Siegel RL, Torre LA, Jemal A (2018). Global cancer statistics 2018: GLOBOCAN estimates of incidence and mortality worldwide for 36 cancers in 185 countries. CA Cancer J Clin.

[2] UNAIDS. UNAIDS data 2019. p. 2019.

[3] Grulich AE, van Leeuwen MT, Falster MO, Vajdic CM (2007). Incidence of cancers in people with HIV/AIDS compared with immunosuppressed transplant recipients: a meta-analysis. Lancet..

[4] Chaturvedi AK, Madeleine MM, Biggar RJ, Engels EA. Risk of Human Papillomavirus – Associated Cancers Among Persons With AIDS. 2009;101(16). 10.1093/jnci/djp205 PMC2728745 19648510

[5] Abraham AG, Strickler HD, Jing Y, Gange SJ, Timothy R, Moore RD (2014). Invasive cervical cancer risk among HIV-infected women: a north American multicohort collaboration prospective study. J Acquir Immune Defic Syndr.

[6] CDC (1993). 1993 revised classification system for HIV infection and expanded surveillance case definition for AIDS among adolescents and adults. JAMA.

[7] Chesson HW, Dunne EF, Hariri S, Markowitz LE (2014). The estimated lifetime probability of acquiring human papillomavirus in the United States. Sex Transm Dis.

[8] Lei J, Ploner A, Elfström KM, Wang J, Roth A, Fang F (2020). HPV vaccination and the risk of invasive cervical Cancer. N Engl J Med.

[9] PATH (2020). Global HPV Vaccine Introduction Overview. Projected and current national introductions, demonstration/pilot projects, gender-neutral vaccination programs, and global HPV vaccine introduction maps (2006-2023).

[10] World Health Organization. Guide to Introducing Hpv Vaccine Into National Immunization Programmes. World Heal Organ. 2016;104 Available from: www.who.int/immunization/documents.

[11] Oshman LD, Davis AM (2020). Human papillomavirus vaccination for adults: updated recommendations of the advisory committee on immunization practices (ACIP). JAMA.

[12] Rodríguez AC, Schiffman M, Herrero R, Wacholder S, Castle PE, Solomon D (2008). Rapid clearance of human papillomavirus and implications for clinical focus on persistent infections. J Natl Cancer Inst.

[13] Plummer M, Schiffman M, Castle PE, Maucort-Boulch D, Wheeler CM (2007). A 2-year prospective study of human papillomavirus persistence among women with a cytological diagnosis of atypical squamous cells of undetermined significance or low-grade squamous intraepithelial lesion. J Infect Dis.

[14] Franco EL, Villa LL, Sobrinho JP, Prado JM, Rousseau MC, Désy M (1999). Epidemiology of acquisition and clearance of cervical human papillomavirus infection in women from a high-risk area for cervical cancer. J Infect Dis.

[15] Muñoz N, Méndez F, Posso H, Molano M, Van Den Brule AJC, Ronderos M (2004). Incidence, duration, and determinants of cervical human papillomavirus infection in a cohort of Colombian women with normal cytological results. J Infect Dis.

[16] Schiffman M, Castle PE, Jeronimo J, Rodriguez AC, Wacholder S (2017). Human papillomavirus and cervical cancer. Lancet.

[17] Castle PE, Rodríguez AC, Burk RD, Herrero R, Wacholder S, Alfaro M (2009). Short term persistence of human papillomavirus and risk of cervical precancer and cancer: population based cohort study. BMJ..

[18] Kjær SK, Frederiksen K, Munk C, Iftner T (2010). Long-term absolute risk of cervical intraepithelial neoplasia grade 3 or worse following human papillomavirus infection: role of persistence. J Natl Cancer Inst.

[19] Ahdieh L, Klein RS, Burk R, Cu-Uvin S, Schuman P, Duerr A (2001). Prevalence, incidence, and type-specific persistence of human papillomavirus in human immunodeficiency virus (HIV)-positive and HIV-negative women. J Infect Dis.

[20] Banura C, Sandin S, Van Doorn LJ, Quint W, Kleter B, Wabwire-Mangen F (2010). Type-specific incidence, clearance and predictors of cervical human papillomavirus infections (HPV) among young women: a prospective study in Uganda. Infect Agent Cancer.

[21] Blitz S, Baxter J, Raboud J, Walmsley S, Rachlis A, Smaill F (2013). Evaluation of HIV and highly active antiretroviral therapy on the natural history of human papillomavirus infection and cervical cytopathologic findings in HIV-positive and high-risk HIV-negative women. J Infect Dis.

[22] Mukanyangezi MF, Rugwizangoga B, Manzi O, Rulisa S, Hellstrand K, Tobin G (2019). Persistence rate of cervical human papillomavirus infections and abnormal cytology in Rwanda. HIV Med.

[23] Koshiol JE, Schroeder JC, Jamieson DJ, Marshall SW, Duerr A, Heilig CM (2006). Time to clearance of human papillomavirus infection by type and human immunodeficiency virus serostatus. Int J Cancer.

[24] Adebamowo Sally N, Famooto A, Dareng EO, Olawande O, Olaniyan O, Offiong R, Adebamowo CA. Clearance of type-specific, low-risk, and high-risk cervical human papillomavirus infections in HIV-negative and HIV-positive women. J Glob Oncol. 2018;2018(4):1–12. 10.1200/JGO.17.00129 PMC6223532 30125130

[25] Kojic EM, Conley L, Bush T, Cu-Uvin S, Unger ER, Henry K (2018). Prevalence and incidence of anal and cervical high-risk human papillomavirus (HPV) types covered by current HPV vaccines among HIV-infected women in the SUN study. J Infect Dis.

[26] Phanuphak N, Teeraananchai S, Hansudewechakul R, Gatechompol S, Chokephaibulkit K, Dang HLD (2020). Incidence and persistence of high-risk Anogenital human papillomavirus infection among female youth with and without perinatally acquired human Immunodefiency virus infection: a 3-year observational cohort study. Clin Infect Dis.

[27] Rowhani-Rahbar A, Hawes SE, Sow PS, Toure P, Feng Q, Dem A (2007). The impact of HIV status and type on the clearance of human papillomavirus infection among Senegalese women. J Infect Dis.

[28] Adler D, Wallace M, Bennie T, Abar B, Sadeghi R, Meiring T (2015). High risk human papillomavirus persistence among HIV-infected young women in South Africa. Int J Infect Dis.

[29] Thorsteinsson K, Ladelund S, Storgaard M, Katzenstein TL, Johansen IS, Pedersen G (2019). Persistence of cervical high-risk human papillomavirus in women living with HIV in Denmark - the SHADE. BMC Infect Dis.

[30] Konopnicki D, Manigart Y, Gilles C, Barlow P, De Marchin J, Feoli F (2013). Sustained viral suppression and higher CD4+ T-cell count reduces the risk of persistent cervical high-risk human papillomavirus infection in HIV-positive women. J Infect Dis.

[31] Strickler HD, Burk RD, Fazzari M, Anastos K, Minkoff H, Massad LS (2005). Natural history and possible reactivation of human papillomavirus in human immunodeficiency virus-positive women. J Natl Cancer Inst.

[32] McClymont E, Lee M, Raboud J, Coutlée F, Walmsley S, Lipsky N (2020). Prevalent and persistent oncogenic HPV types in a cohort of women living with HIV prior to HPV vaccination. Int J Gynecol Obstet.

[33] Kelly HA, Sawadogo B, Chikandiwa A, Segondy M, Gilham C, Lompo O (2017). Epidemiology of high-risk human papillomavirus and cervical lesions in African women living with HIV/AIDS: effect of anti-retroviral therapy. Aids..

[34] Gui LIU, Monisha SHARMA, Nicholas TANRB (2018). HIV-positive women have higher risk of HPV infection, precancerous lesions, and cervical cancer: a systematic review and meta-analysis. AIDS..

[35] Adebamowo SN, Olawande O, Famooto A, Dareng EO, Offiong R, Adebamowo CA (2017). Persistent low-risk and high-risk human papillomavirus infections of the uterine cervix in HIV-negative and HIV-positive women. Front Public Health.

[36] Murenzi G, Kanyabwisha F, Murangwa A, Kubwimana G, Mutesa L, Burk RD (2020). Twelve-year trend in the prevalence of high-risk human papillomavirus infection among rwandan women living with HIV. J Infect Dis.

[37] Murenzi G, Dusingize J-C, Rurangwa T, Sinayobye J d’A, Munyaneza A, Murangwa A (2018). Protocol for the study of cervical cancer screening technologies in HIV-infected women living in Rwanda. BMJ Open.

[38] Atila B (2019). Genotype 15 high risk HPV by fluorescent detection.

[39] Mũnoz N, Hernandez-Suarez G, Méndez F, Molano M, Posso H, Moreno V (2009). Persistence of HPV infection and risk of high-grade cervical intraepithelial neoplasia in a cohort of Colombian women. Br J Cancer.

[40] Joura EA, Giuliano AR, Iversen O-E, Bouchard C, Mao C, Mehlsen J (2015). A 9-Valent HPV vaccine against infection and intraepithelial Neoplasia in women. N Engl J Med.

