# Supplementary material for: Type-specific persistence, clearance and incidence of high-risk HPV among screen-positive Rwandan women living with HIV
Source: Infect Agent Cancer. 2021 Feb 19;16:16. doi: 10.1186/s13027-021-00355-6 (PMC7893720; doi:10.1186/s13027-021-00355-6)
Supplement: Supplementary file 1 — Additional file 1: Supplemental Table 1. Comparison of screen-positive women included in this analysis and those not included. [file 13027_2021_355_MOESM1_ESM.docx]

**Supplemental table**: Comparison of screen-positive women included in this analysis and those not included

| **Characteristic** | | **Women included in this analysis** | **Women not included in this analysis** | **p*** |
| --- | --- | --- | --- | --- |
|  |  | **n=298** | **n=1,116** |  |
|  |  | **n (%col)** | **n (%col)** |  |
| **Age (Years)** | |  |  |  |
|  | Mean±SD | 38.6±6.2 | 40.4±6.6 | <0.01 |
|  | Median (IQR) | 37 (34-43) | 40 (35-45) |  |
| **Age at Sexual Initiation (Years)** | |  |  |  |
|  | <18 | 180 (60.4) | 406 (36.4) | <0.01 |
|  | ≥18 | 118 (39.6) | 710 (63.6) |  |
| **Number of Sexual Partners (Lifetime)** | |  |  |  |
|  | ≤5 | 245 (82.2) | 909 (81.4) | 0.87 |
|  | ≥6 | 53 (17.8) | 205 (18.4) |  |
|  | Missing |  | 2 (0.2) |  |
| **Number of Sexual Partners (Last 6 Months)** | |  |  |  |
|  | ≤5 | 285 (95.6) | 1,070 (95.9) | 0.50 |
|  | ≥6 | 8 (2.7) | 20 (1.8) |  |
|  | Missing | 5 (1.7) | 26 (2.3) |  |
| **Number of Children** | |  |  |  |
|  | <5 | 230 (77.2) | 880 (78.8) | 0.53 |
|  | ≥5 | 68 (22.8) | 236 (21.2) |  |
| **CD4 (per mm^3^) (Screening Visit)** | |  |  |  |
|  | ≥500 (ref) | 146 (49.0) | 603 (54.0) | 0.51 |
|  | 350-499 | 64 (21.5) | 232 (20.8) |  |
|  | 200-349 | 48 (16.1) | 146 (13.1) |  |
|  | <200 | 25 (8.4) | 82 (7.4) |  |
|  | Missing | 15 (5.0) | 53 (4.7) |  |
| **On Anti-Retrovirals** | |  |  |  |
|  | No | 3 (1.0) | 17 (1.5) | 0.53 |
|  | Yes | 293 (98.3) | 1,082 (97.0) |  |
|  | Missing | 2 (0.7) | 17 (1.5) |  |
| **HIV Viral Load (Screening Visit)** | |  |  |  |
|  | Undetectable | 236 (79.2) | 901 (80.7) | 0.73 |
|  | Detectable | 28 (9.4) | 104 (9.3) |  |
|  | Missing | 34 (11.4) | 111 (10.0) |  |
| **VIA Result (Screening Visit)** | |  |  |  |
|  | Negative | 228 (76.5) | 718 (64.3) | <0.01 |
|  | Positive | 68 (22.8) | 385 (34.5) |  |
|  | Missing | 2 (0.7) | 13 (1.2) |  |
| **hrHPV positivity (Screening Visit-Xpert test)** | |  |  |  |
|  | Negative | 47 (15.8) | 251 (22.5) | 0.01 |
|  | Positive | 251 (84.2) | 861 (77.1) |  |
|  | Missing |  | 4 (0.4) |  |
| **Colposcopic Impression** | |  |  |  |
|  | Low-Grade | 266 (89.3) | 1,064 (95.3) | <0.01 |
|  | High-Grade | 25 (8.4) | 47 (4.2) |  |
|  | Missing | 7 (2.4) | 5 (0.5) |  |

*Fisher’s Exact to compare screen-positive women included in this analysis vs. those not included
